# Activation of adenosine A3 receptor reduces early brain injury by alleviating neuroinflammation after subarachnoid hemorrhage in elderly rats

**DOI:** 10.18632/aging.202178

**Published:** 2020-11-30

**Authors:** Peng Li, Xiaojun Li, Peng Deng, Dandan Wang, Xuehong Bai, Yujie Li, Chunxia Luo, Karine Belguise, Xiaobo Wang, Xinchuan Wei, Zhengyuan Xia, Bin Yi

**Affiliations:** 1 Department of Anesthesia, Southwest Hospital, Third Military Medical University, Chongqing, China; 2 Department of Anesthesia, Sichuan Academy of Medical Sciences and Sichuan Provincial People’s Hospital, Chengdu, China; 3 Department of Neurology, Southwest Hospital, The Third Military Medical University, Chongqing, China; 4 Laboratoire de Biologie Cellulaire et Moléculaire du Contrôle de la Prolifération (LBCMCP), University P. Sabatier, Toulouse Cedex 9, France; 5 Department of Anesthesiology, University of Hong Kong, Hong Kong SAR, China; 6 Department of Cerebrovascular Diseases, The Second Affiliated Hospital of Zhengzhou University, Zhengzhou, China

**Keywords:** subarachnoid hemorrhage, microglial polarization, adenosine A3 receptor, anti-inflammation

## Abstract

The incidence of subarachnoid hemorrhage (SAH) and hazard ratio of death increase with age. Overactivation of microglia contributes to brain damage. This study aimed to investigate the effects of A3 adenosine receptors (A3R) activation on neurofunction and microglial phenotype polarization in the context of SAH in aged rats. The A3R agonist (CI-IB-MECA) and antagonist (MRS1523) were used in the SAH model. Microglia were cultured to mimic SAH in the presence or absence of CI-IB-MECA and/or siRNA for A3R. The neurofunction and status of the microglial phenotype were evaluated. The P38 inhibitor SB202190 and the STAT6 inhibitor AS1517499 were used to explore the signaling pathway. The results showed that SAH induced microglia to polarize to the M(LPS) phenotype both *in vivo* and *in vitro*. CI-IB-MECA distinctly skewed microglia towards the M(IL-4) phenotype and ameliorated neurological dysfunction, along with the downregulation of inflammatory cytokines. Knockdown of A3R or inhibition of P38 and/or STAT6 weakened the effects of CI-IB-MECA on microglial phenotypic shifting. Collectively, our findings suggest that activation of A3R exerted anti-inflammatory and neuroprotective effects by regulating microglial phenotype polarization through P38/STAT6 pathway and indicated that A3R agonists may be a promising therapeutic options for the treatment of brain injury after SAH.

## INTRODUCTION

Subarachnoid hemorrhage (SAH) is a common cerebrovascular disease with high morbidity and mortality. In populations with a mean age of 35 years, calculated incidence of SAH was 8.6 per 100000, and the incidence was 1.06 times higher for every year with the increase in mean age [1]. The risk of death increases by 6% per year of age and the risk for adverse outcome by 11% per year of age in elderly patients [2].

Accumulated evidence has shown that early brain injury (EBI) induced by microglia activation contributes to poor outcomes after SAH [3, 4]. Microglia are resident macrophages in the central nervous system and exhibit multiple functions depending on the stage of life and context of health or disease [5]. In an attempt to structure the complexity of microglial activation, an early categorization was applied to classify activated microglia into classical activation (M1) or alternative activation (M2) [6]. The M1 microglia promotes inflammation by the production of pro-inflammatory cytokines (TNF-α, IL-6, and IL-1β), chemokines, and reactive oxygen species. In contrast, M2 microglia facilitates debris clearance, wound healing, and restoration of brain tissue homeostasis by the production of protective/trophic factors (Arg1, IL-4, and IL-10) [6, 7]. Therefore, suppressing the inflammation mediated by activated microglia would be a promising therapeutic target to prevent neurological impairment in SAH [8]. It is rather remarkable that microglia seem to obtain an age-related immune-activation, which may contribute to the pathology of neurodegenerative diseases [6]. Age significantly affects the capacity of the brain to elicit polarized microglial responses, with a bias toward M1 and away from M2 activation states [9]. However, study also showed that aging alone—without any additional inflammatory trigger—does not necessarily result in a pro-inflammatory microglia activation [10]. Considering the complexity of microglia in aging and SAH, it is urgent to study the role of microglia in EBI after SAH in the elderly. In addition, emerging evidence suggests that microglia display differences in their functions determined both by their milieu and by the unique properties these cells possess [5]. Hence the dichotomy polarization of microglia is considered outdated and it is necessary to refine the M1/M2 nomenclature by adding the triggering stimulus as an abbreviation to the M1 or M2 classification [6, 11]. In accordance to the defined terminology, we are here calling M1-like microglia M(LPS) and M2-like microglia M(IL-4) [11, 12].

Adenosine (ADO) is a metabolic product of endogenous adenosine triphosphate (ATP), and it works as an important neuromodulator in the central nervous system (CNS). In pathological state, such as ischemia and hypoxia, the extracellular concentration of ADO increases sharply [13]. As one of four adenosine receptors, A3 adenosine receptor (A3R) is upregulated in activated inflammatory cells and has anti-inflammatory effects in various animal models [14, 15]. A large number of studies have shown that A3R participates in the regulation of the activation of inflammatory immune cells, such as microglia, neutrophils and T cells, and thus controls the release of various inflammatory mediators [16–19]. Although A3R activation mediates neuroprotective effects, it has also been shown to induce apoptosis in cancer cells and optic nerve oligodendrocytes [20]. In addition, A3R have also been identified in neurons and astrocytes in brain [21]. Studies in this field have focused on the role of A3R in synaptic modulation and apoptosis of neurons, but the results are rather contradictory and have been a matter of intense debate [22, 23].

Our previous study found that the A3R activation can reduce cerebral edema and protect neural function in adult rat experimental SAH models, which may be related to the inhibition of microglial activation [24]. However, how microglia function is regulated and how A3R plays a role in the SAH model of aging rodents remain to be explored and clarified. In this study, we hypothesize that activation of A3R can modulate microglial function and reduce EBI by regulating the inflammatory response after subarachnoid hemorrhage.

## RESULTS

### General information and neurological score

**Figure 1:**
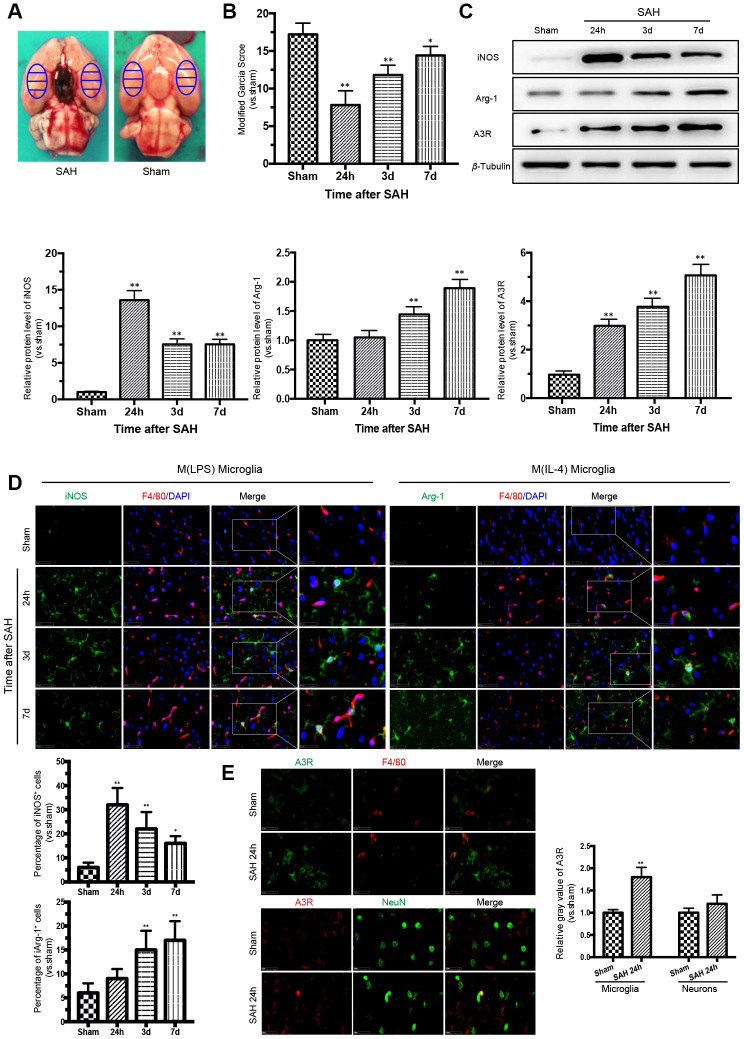
**Time courses of the neurological scores, phenotypic characteristics of microglia and expression of adenosine A3 receptor after subarachnoid hemorrhage (SAH).** (**A**) Representative images showing blood clots deposited around the circle of Willis and schematic representations of the areas for analysis. (**B**) The modified Garcia score was significantly reduced after SAH, with the lowest point at 24 h. (**C**) Western blot analysis showing the protein expression levels of nitric-oxide synthase (iNOS), arginase-1 (Arg-1), and adenosine A3 receptor (A3R) at 24 h, 3 days, and 7 days after SAH. (**D**) Immunofluorescence staining showing the expression of iNOS and Arg-1 in the cerebral cortex. iNOS- and Arg-1-positive cells were analyzed quantitatively. (**E**) A3R staining was observed in the cerebral cortex at 24h after SAH. F4/80 and NeuN was respectively used as a microglia and neurons marker. The relative gray value of A3R were quantified. *n*=5 in each group. Data are shown as the mean ± SD. *p < 0.05 and **p < 0.01 versus sham group.

There was no death of rats in the sham group. Of the 82 rats subjected to SAH, 13 rats (15.8%) died within 24 h of SAH, and 9 rats were excluded due to mild SAH. The samples adjacent to the subarachnoid blood clots that deposited at the bilateral basal temporal lobes were taken for analysis (Figure 1A). The modified Garcia score was severely impaired after SAH, with the lowest point at 24 h (Figure 1B).

### Phenotypic conversion of microglia after SAH was accompanied by A3R upregulation

To detect the M(LPS)- and M(IL-4)-associated markers in microglia in the cerebral cortex after SAH, western blot analysis and immunofluorescence double staining were performed to assess the expression of iNOS and Arg-1. The expression of iNOS was highest at 24 h after SAH and then decreased gradually, while the expression of Arg-1 increased with time, reaching a peak at 7 days after SAH. Moreover, the trend in A3R expression was consistent with that of Arg-1 (Figure 1C). The immunofluorescence staining of iNOS and Arg-1 indicated that microglia act as a double-edged sword in the process of SAH (Figure 1D). Immunostaining confirmed that A3R was upregulated in both the neurons and the microglia at 24h after SAH (Figure 1E). Compared with the sham group, the A3R was slightly upregulated in neurons (*p*>0.05), but significantly increased in microglia (*p*<0.01) in rats with SAH. These results indicate that A3R may be involved in the phenotypic conversion of microglia after SAH.

### The A3R agonist ameliorated neuronal apoptosis and improved neurobehavioral outcomes by regulating inflammatory cytokines after SAH

**Figure 2:**
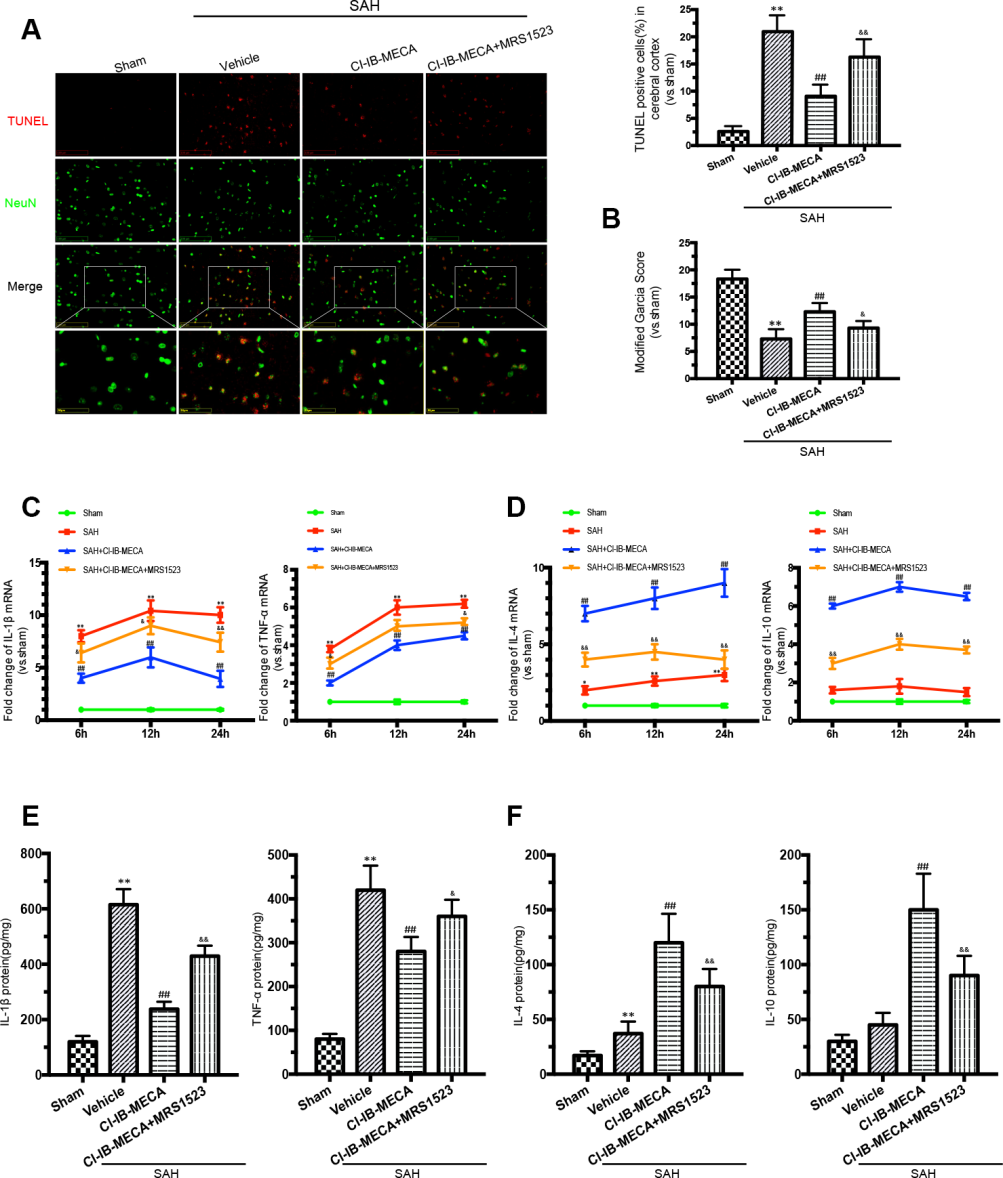
**The A3R agonist ameliorated neuronal apoptosis and improved neurobehavioral outcomes by regulating neuroinflammatory after SAH.** (**A**) Apoptotic neurons and percentage of TUNEL-positive cells in the cerebral cortex 24 h after SAH. NeuN was used as a neuron marker. (**B**) Neurologic score was performed 24 h after SAH. (**C**, **D**) The changes of IL-1β, TNF-α, IL-4 and IL-10 mRNA at 6 h, 12 h and 24 h after SAH. (**E**, **F**) The inflammatory cytokines was measured by enzyme-linked immunosorbent assay at 24 h after SAH. *n*=5 in each group. Values are shown as the mean ± SD. *p < 0.05 and **p < 0.01, versus sham group; ## *p* < 0.01, versus SAH group; & *p* < 0.05 and && *p* < 0.01 versus SAH+CI-IB-MECA group.

Based on the above results and also previously reported findings, microglial activation and behavioral impairment peaked at 24 h after SAH; thus, rats that received different treatments were sacrificed 24 h after SAH for the immunofluorescence staining, western blot and PCR analyses [25]. To investigate the role of the A3R agonist in SAH, CI-IB-MECA was administered twice before SAH. Neurological functions were significantly improved by pre-treatment with CI-IB-MECA before SAH when compared with those of the SAH group (Figure 2B). Oppositely, this neuroprotective effect was impaired by the administration of MRS1523, the A3R antagonist. To further verify the neuroprotective effect of CI-IB-MECA, TUNEL staining for apoptosis assay was performed 24 h after SAH. NeuN was used to mark neurons. Compared with the Sham group, there was a significant increase in apoptotic neurons in the SAH group. With the administration of CI-IB-MECA, neuronal apoptosis was dramatically attenuated. In contrast, the number of apoptotic neurons significantly increased with the use of MRS1523 (Figure 2A). Next, we characterized the mRNA and protein expression of proinflammatory and anti-inflammatory cytokines, both of which have been implicated in the pathology of SAH. As shown in Figure 2C, the proinflammatory cytokines IL-1β and TNF-α were elevated after SAH. CI-IB-MECA treatment markedly decreased the expression of IL-1β and TNF-α, which were partially reversed by MRS1523. Instead, the concentrations of the anti-inflammatory cytokines interleukin 4 (IL-4) and interleukin 10 (IL-10) were significantly increased following the activation of A3R as compared with those of the SAH group (Figure 2D). Furthermore, enzyme-linked immunosorbent assay also confirmed that CI-IB-MECA induced IL-4 and IL-10, but reduced IL-1β and TNF-α at 24h after SAH (Figure 2E, 2F). Taken together, these results demonstrated that A3R agonists improved neurobehavioral outcomes after SAH by reducing cortex neuronal apoptosis and regulating inflammatory cytokines.

### Activation of A3R modulated the microglial phenotype after SAH *in vivo*

**Figure 3:**
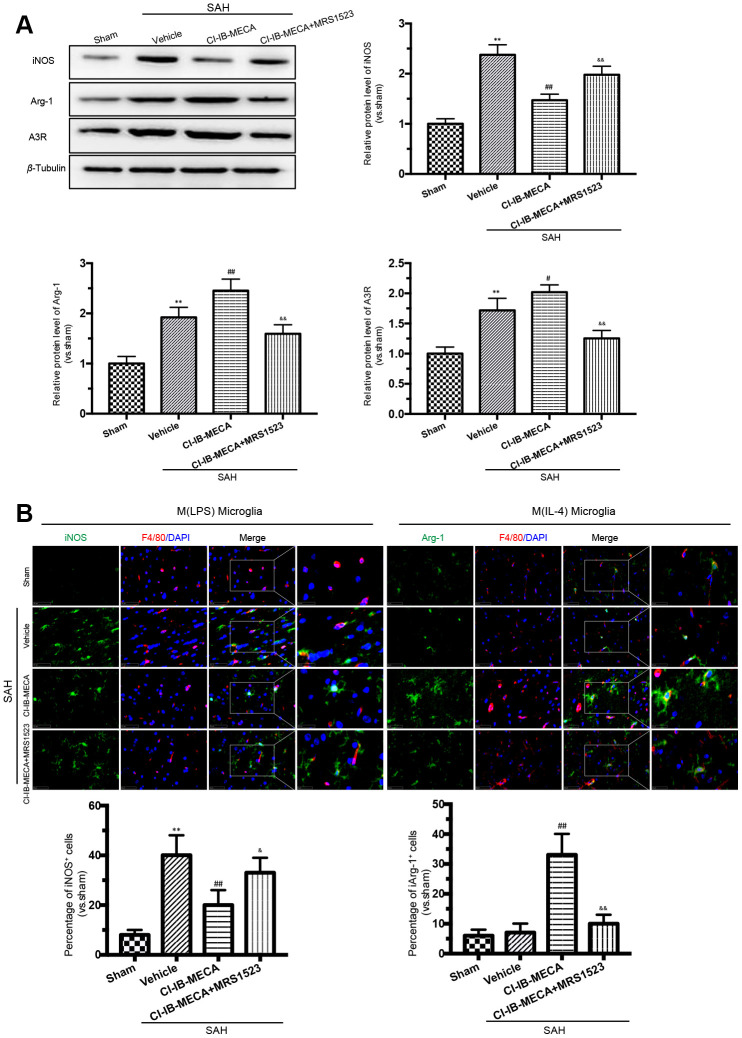
**A3R agonist CI-IB-MECA promotes microglia towards to the M(IL-4) phenotype in rat 24 h after SAH.** (**A**) Western blot analysis showing the expression of iNOS and Arg-1 after injection of CI-IB-MECA with or without MRS1523 24 h after SAH. A3R protein expression increased 24 h after SAH and was further increased after CI-IB-MECA treatment, which was partially reversed by the A3R antagonist MRS1523. (**B**) Immunofluorescence staining showing the M(LPS)-associated marker iNOS or the M(IL-4)-associated marker Arg-1 in the cerebral cortex 24 h after SAH. Both M(LPS) and M(IL-4) microglia were quantified. *n*=5 in each group. Values are shown as the mean ± SD. **p < 0.01, versus sham group; #*p* < 0.05 and ## *p* < 0.01, versus SAH group; & *p* < 0.05 and && *p* < 0.01 versus SAH+ CI-IB-MECA group.

As shown in Figure 3A, 3B, activation of A3R resulted in a decrease in iNOS expression in the cerebral cortex, while this effect was weakened when the animals were pretreated with the A3R antagonist MRS1523. In contrast, the A3R effect on the expression of the M (IL-4) microglial marker Arg-1 showed the opposite pattern. Treatment with CI-IB-MECA increased Arg-1 expression, which was weakened by MRS1523 (Figure 3A, 3B). In addition, there was an enhancement of the A3R protein expression after SAH, which was further increased in the presence of CI-IB-MECA treatment (Figure 3A). In summary, these results suggested that the upregulation and activation of A3R might contribute to the switch in microglial phenotypes.

### CI-IB-MECA alleviated the OxyHb-induced neuronal damage via “direct” and “indirect” routes, mainly by modulating microglial phenotype polarization *in vitro*

**Figure 4:**
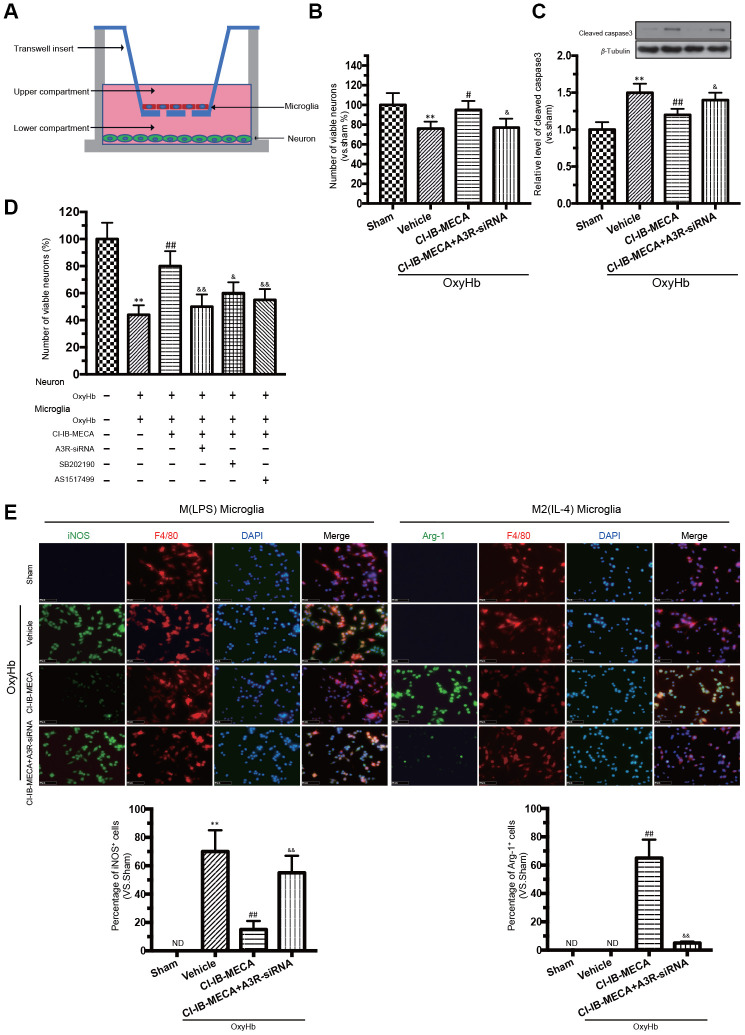
**A3R agonist alleviated oxyhemoglobin (OxyHb) induced neuronal apoptosis through “direct” and “indirect” effects, accompanied by a significant microglia M(IL-4) polarization *in vitro*.** (**A**) Mimetic diagram of the Transwell co-culture system. (**B**, **C**) The effect of A3R agonist on neuronal apoptosis and expression of caspase 3. (**D**) Effects of CI-IB-MECA and P38 and STAT6 inhibitors on neuronal apoptosis with a Transwell co-culture system. (**E**) Microglia polarized to the classically activated phenotype after the treatment with OxyHb, and CI-IB-MECA increased the number of Arg-1-positive and decreased the iNOS-positive microglia *in vitro*. Quantitative analysis of the percentage of iNOS- and Arg-1-positive cells in different groups under the indicated treatments. *n*=5 in each group. Values are shown as the mean ± SD. **p < 0.01 versus sham group; ## *p* < 0.01 versus OxyHb group; & *p* < 0.05 and && *p* < 0.01 versus OxyHb +CI-IB-MECA group.

Since our results gained from the *in vivo* studies could be due to the effects coming from both neurons and microglia, a co-cultured *in vitro* system was used to discriminate the respective effect from either type of cells. This co-cultured *in vitro* system has been demonstrated to simulate the effect of different cells *in vivo* [26, 27]. Here, transwell culture system was used to examine the “direct” and “indirect” effects of CI-IB-MECA on primary neurons stimulated by OxyHb (Figure 4A). To determine the direct effect, only neurons were cultured with OxyHb. OxyHb induced apoptosis in the primary neurons by reducing cell vitality (76%±7%, *p*=0.0048). By contrast, CI-IB-MECA directly reduced the OxyHb-induced neuron apoptosis and caspase-3 expression (Figure 4B, 4C). To determine the combined effects from both neurons and microglia, microglia and neurons were separately incubated with OxyHb for 24 h, and then both pre-treated cells were cultured together in the tanswell system for another 24 h. Indeed, microglia pre-treated with OxyHb significantly exacerbated the apoptosis of neurons (44%±8%, *p*<0.0001), suggesting that the activation of microglia was an important factor to further increase neuronal injury. In contrast, pre-treat the microglia with CI-IB-MECA significantly mitigated the neuronal injury (Figure 4D). Because we showed above that CI-IB-MECA can switch the phenotypic polarization of microglia from the M(LPS) to the M(IL-4) type, we next investigated whether the role of microglia in neuronal injury is related to this phenotypic polarization. As shown in Figure 4E, indeed, iNOS-positive cells increased significantly after the OxyHb incubation. This result confirmed that CI-IB-MECA suppressed M(LPS) phenotype polarization and promoted BV-2 cells towards to the M(IL-4) phenotype. In contrast, in the A3R siRNA group, the knockdown of A3R abolished the CI-IB-MECA-mediated microglial phenotype polarization (Figure 4E). Therefore, our results suggested that activation of A3R could alleviate the neuron apoptosis by directly protecting neurons and indirectly regulating the microglia phenotypic polarization, the latter of which seemed to play another important role to further enhance the apoptosis of neurons.

### The transcription factors P38, STAT6 and NF-κB were involved in the CI-IB-MECA- induced microglial phenotype polarization

**Figure 5:**
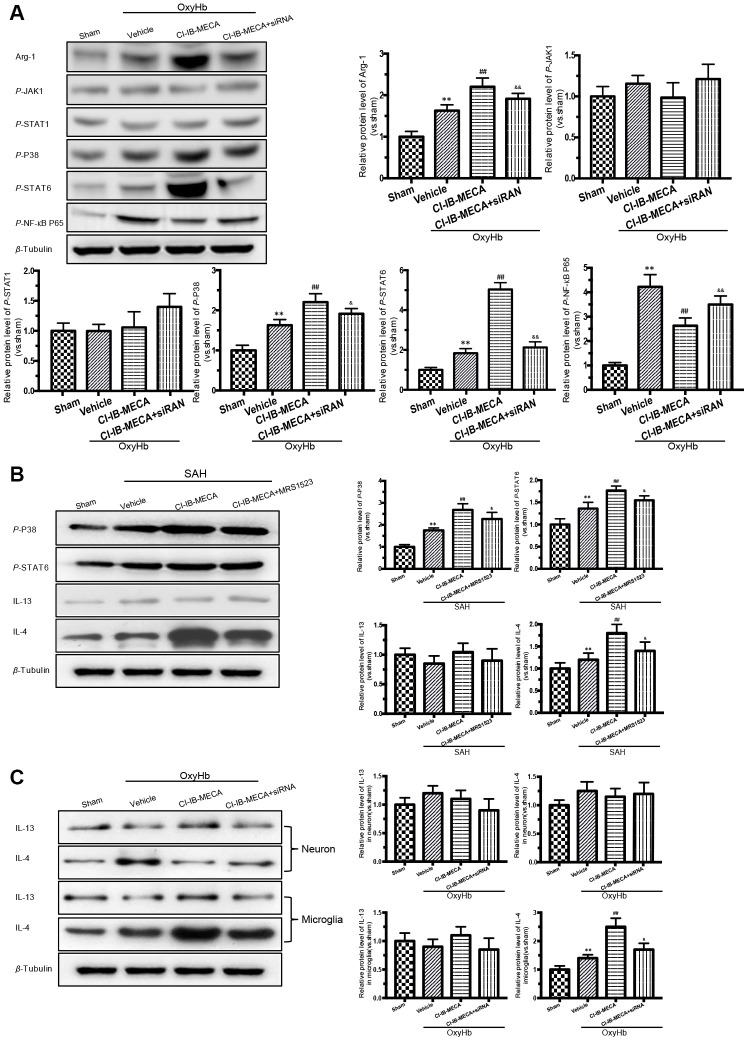
**Activation of A3R induced the expression of Arg-1 *in vitro*, and enhanced the activation of P38, STAT6 both *in vitro* and *in vivo*.** (**A**) Western blot analysis showing the expression of Arg-1 and phosphorylated JAK1, STAT1, P38, STAT6 and NF-κB P65 24 h after OxyHb treatment. The protein levels were quantified. (**B**) Effects of the A3R agonist (CI-IB-MECA) and antagonist (MRS1523) on the P38/STAT6 pathway, IL-13 and IL-4(STAT6 activator) *in vivo* 24 h after SAH. Western blot analysis showing the protein of phosphorylated P38 (p-P38), STAT6 (p-STAT6), IL-13 and IL-4 under the indicated treatments. (**C**) Effect of CI-IB-MECA on the expression of IL-13 and IL-4, in neuron or microglia *in vitro*. β-Tubulin was used as a loading control. *n*=5 in each group. Data are shown as the mean ± SD. **p < 0.01, versus sham group; ## *p* < 0.01, versus OxyHb group or SAH group; & *p* < 0.05 and && *p* < 0.01 versus OxyHb +CI-IB-MECA group or SAH+CI-IB-MECA group.

The known main transcription factors involved in the macrophage polarization include Janus kinases (JAKs), signal transducers and activators of transcription (STATs) and nuclear factor kappa-B (NF-κB) [28]. A3R activation has also been known to exert the anti-inflammatory effects by regulating P38 [16]. We thus evaluated the effect of the A3R agonist on the expression of transcription factors in the cultured microglia stimulated with OxyHb. The western blot results showed that stimulation with OxyHb increased the levels of Arg-1 expression, p-P38, p-STAT6, and p-NF-κB p65 but had no effect on the expression levels of JAK1 and STAT1 (Figure 5A). The A3R agonist further elevated the levels of Arg-1, p-P38, and p-STAT6 but suppressed p-NF-κB p65 (Figure 5A). However, knockdown of A3R partially eliminated the effect of CI-IB-MECA. Next, we explored the effect of CI-IB-MECA on the activation of p-P38 and p-STAT6 *in vivo*. As shown in Figure 5B, the activation of p-P38 and p-STAT6 were increased in the cortex 24 h after SAH compared with that of the sham group, and CI-IB-MECA treatment further increased the levels of p-P38 and p-STAT6. Furthermore, administration of MRS1523 decreased the activation of p-P38 and p-STAT6 *in vivo* compared with that of the CI-IB-MECA group (Figure 5B). These data suggested that activation of A3R upregulated the expression of Arg-1 by activating P38 and STAT6. In addition, the best known activators of STAT6 are IL-13 and IL-4, which is typically upregulated upon neuronal injury [29, 30]. To further demonstrate that the A3R mediated microglial polarization through the P38/STAT pathway, we measured the expression of IL-13 and IL-4 in the cortex. Neither SAH nor CI-IB-MECA increased the expression of IL-13. By contrast, the expression of IL-4 increased after SAH, and the activation of A3R further enhanced its expression (Figure 5B). Because both microglia and neurons can produce these two activators upstream of STAT6 during brain injury, we measured the changes of IL13 and IL4 in neurons and microglia *in vitro*. Neither OxyHb nor CI-IB-MECA induced the expression of IL13 and IL4 in the primary cultured neurons (Figure 5C). However, OxyHb increased the IL-4 expression in microglia that was further enhanced by the activation of A3R. Oppositely, the A3R antagonist reversed the effect of CI-IB-MECA on IL-4 (Figure 5C). The results suggested that SAH upregulated the expression of IL-4, which was mainly mediated by microglia rather than by neurons. As an activator of STAT6, IL-4 induces phosphorylation of STAT6, which in turn causes microglia to release IL-4 [27]. The results indicated that the upregulation of IL-4 induced by CI-IB-MECA could trigger a positive feedback to facilitate the microglia phenotypic polarization.

### The A3R agonist modulated the microglial polarization via the P38/STAT6 signaling pathway

**Figure 6:**
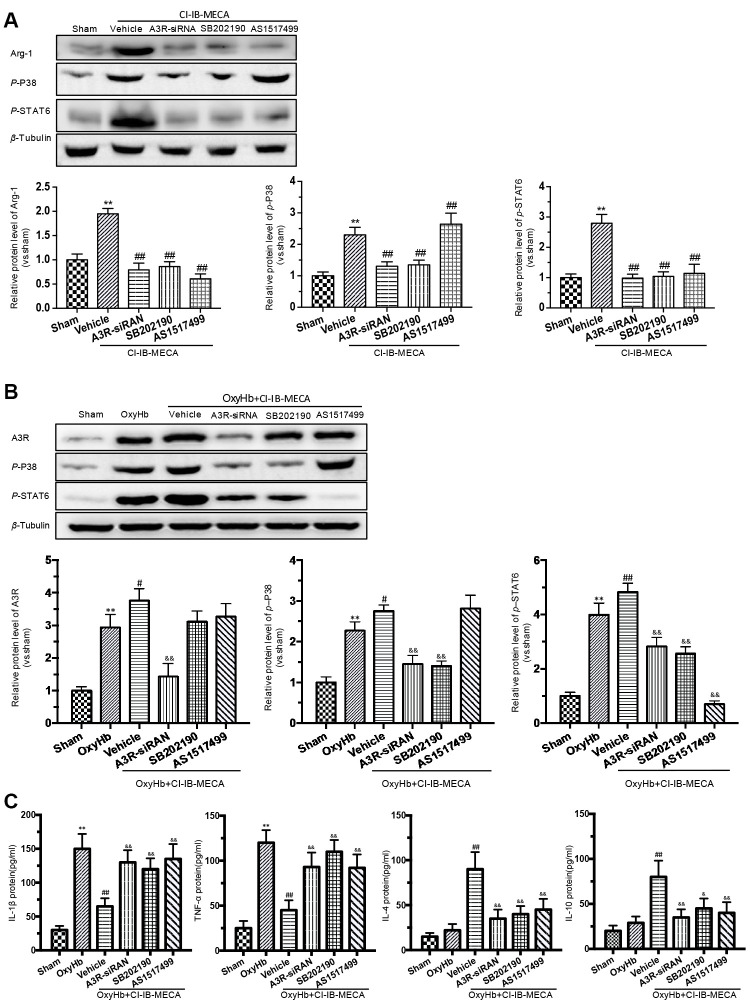
**The effects of A3R agonist CI-IB-MECA on microglial polarization are modulated by the A3R/P38/STAT6 pathway.** (**A**) The relationship between the expression of Arg-1 and the A3R/P38/STAT6 axis. To clarify the changes in P38, STAT6 and Arg-1 expression after activation of the adenosine A3 receptor, microglia were treated with CI-IB-MECA alone. Western blots showing the expression of Arg-1 and phosphorylated P38 and STAT6 after pretreatment with A3R siRNA, the P38 blocker (SB202190) and the STAT6 blocker (AS1517499) in microglia treated with CI-IB-MECA. The expression of Arg-1, p-P38 and p-STAT6 under the indicated treatments was also quantified. (**B**) To further simulate SAH *in vitro* and demonstrate the role of the A3R/P38/STAT6 pathway, microglia were treated with OxyHb for 24 h in the presence or absence of CI-IB-MECA, A3R siRNA, the P38 blocker (SB202190) or the STAT6 blocker (AS1517499). The protein expression of A3R, p-P38 and p-STAT6 was upregulated after OxyHb stimulation and was further upregulated after the treatment with A3R agonists, which was reversed by A3R siRNA. Inhibition of P38 also decreased STAT6 activation but did not increase A3R expression. (**C**) The A3R agonist decreased the release of inflammatory cytokines in microglia treated with OxyHb for 24 h. IL-1β and TNF-α increased after treatment with OxyHb and was suppressed by CI-IB-MECA, which was reversed by blocking A3R, P38 or STAT6. IL-4 and IL-10 increased significantly after activation of A3R and decreased after the inhibition of the A3R/P38/STAT6 axis. *n*=5 in each group. Values are shown as the mean ± SD. **p < 0.01, versus sham group; # *p* < 0.05, versus OxyHb group; ## *p* < 0.01, versus CI-IB-MECA group or OxyHb group; & *p* < 0.05 and && *p* < 0.01 versus OxyHb+CI-IB-MECA group.

A3R activation stimulates the intracellular phosphorylation cascade of the MAPK family, including P38, which is involved in immunocyte M(IL-4) polarization by activating STAT6 [16, 31]. Previous study revealed that IL-4 induces phosphorylation of p38 in macrophages, in addition to STAT-6 and PI3K activation, indicating the involvement of p38 MAPK in the signaling of IL-4 leading to M2-macrophage polarization [31]. Therefore, we hypothesized that the A3R agonist might play a role in regulating the phenotypic transformation of microglia through the A3R/P38/STAT6 pathway. We used CI-IB-MECA to treat microglia in order to assess the effect of A3R activation on the signals of p-P38, p-STAT6 and Arg-1. CI-IB-MECA upregulated the levels of the Arg-1, p-P38, and p-STAT6 (Figure 6A). The increase of Arg-1 was blocked by the A3R siRNA, the P38 inhibitor SB202190 or the STAT6 inhibitor AS1517499, indicating that Arg-1 might lie downstream of the A3R/P38/STAT6 pathway. Consistently, A3R knockdown abolished CI-IB-MECA-mediated increases in p-P38 and p-STAT6 (Figure 6A), suggesting that A3R lies upstream of this pathway. Furthermore, the increase in p-STAT6 was suppressed by the P38 inhibitor SB202190, while the STAT6 inhibitor AS1517499 had no effect on the activation of p-P38, indicating that P38 affects STAT6 phosphorylation, but not vice verse. (Figure 6A). In addition, in order to further verify whether CI-IB-MECA plays a neuroprotective role through the P38 and STAT6 pathway, P38 and STAT6 inhibitors were used in the co-culture system of microglia and neurons. The results demonstrated that inhibition of P38 or STAT6 could reverse the protective effect of CI-IB-MECA on neuron apoptosis (Figure 4D). Collectively, these data demonstrated that A3R activation induced the M(IL-4) microglial polarization via the P38/STAT6 signaling pathway.

### CI-IB-MECA regulated the release of inflammatory cytokines in the OxyHb-stimulated microglia through the A3R/P38/STAT6 signaling pathway

We showed that A3R agonist ameliorated neuronal apoptosis and improved neurobehavioral outcomes by regulating inflammatory cytokines after SAH. Furthermore, we investigated the effect of the A3R/P38/STAT6 pathway on the inflammatory response. CI-IB-MECA administration decreased the expression of IL-1β and TNF-α and increased IL-4 and IL-10 after stimulation with OxyHb (Figure 6C). A3R knockdown, the P38 inhibitor SB202190 and the STAT6 inhibitor AS1517499 all reversed the CI-IB-MECA-mediated effect on the expression of inflammatory cytokines after the OxyHb treatment (Figure 6C). We further explored the effect of OxyHb stimulation on the levels of the A3R/P38/STAT6 signaling pathway. As shown in Figure 6B, CI-IB-MECA amplified the OxyHb-induced increases in A3R, p-P38 and p-STAT6. Collectively, these results demonstrated that the CI-IB-MECA-induced activation of the A3R/P38/STAT6 pathway regulated the release of inflammatory cytokines in OxyHb-stimulated microglia *in vitro*.

## DISCUSSION

Although substantial progress has been made in understanding the complex mechanisms that cause neurological impairment after SAH, the available neuroprotective therapies for SAH remain limited.

Neuroinflammation is considered as a predominant pathological hallmark of brain damage after SAH [32, 33]. During neuroinflammation following SAH, activated microglia are a pivotal source for a plethora of various cytokines and chemokines in the central nervous system [34]. As resident immune cells, microglia are critical for brain development, maintain health, and rapidly adapt their function to the physiological or pathophysiological needs [35]. It is well recognized that microglia display a wide range of reaction states, and the M1/M2 classification is widely prevalent [36, 37]. M1(pro-inflammatory) microglia are associated with brain damage by elevating the expression of inflammatory cytokines. In contrast, M2(anti-inflammatory) microglia are characterized by releasing anti-inflammatory mediators that have neuroprotective functions [38, 39]. However, emerging evidence suggests that microglia differ in function not exclusively because of their milieu, but also because of their unique properties [5]. Taken into account the complex plasticity (reaction states) and diversity (subtypes) of microglia, much attention has been paid to the reconsideration of the description of microglial phenotypes based on their macrophage attributes M1 and M2 [35, 40]. Although the M1/M2 classification still widely used in the emerging published articles [41–43], it is necessary to refine the M1/M2 nomenclature by adding the triggering stimulus as an abbreviation to the M1 or M2 classification [6, 11]. Hence we are here calling M1-like microglia M(LPS) and M2-like microglia M(IL-4) [11, 12]. Not only does SAH cause severe neuroinflammatory, but microglia also reveal age-related functional and phenotypic changes. Microarray gene expression analyses showed that anti-inflammatory genes in microglia are reduced in aged individuals [44]. In the absence of pathology, aged microglia express higher pro-inflammatory genes or antigen presenting markers and produce more pro-inflammatory cytokines; while anti-inflammatory cytokines and microglial activation inhibitory factors are down-regulated [35]. However, study also shows that aging alone—without any additional inflammatory trigger—does not necessarily result in a pro-inflammatory microglia activation [10]. Consequently, it is undeniable that microglia are more likely to mediate neuroinflammatory in aged individuals.

**Figure 7:**
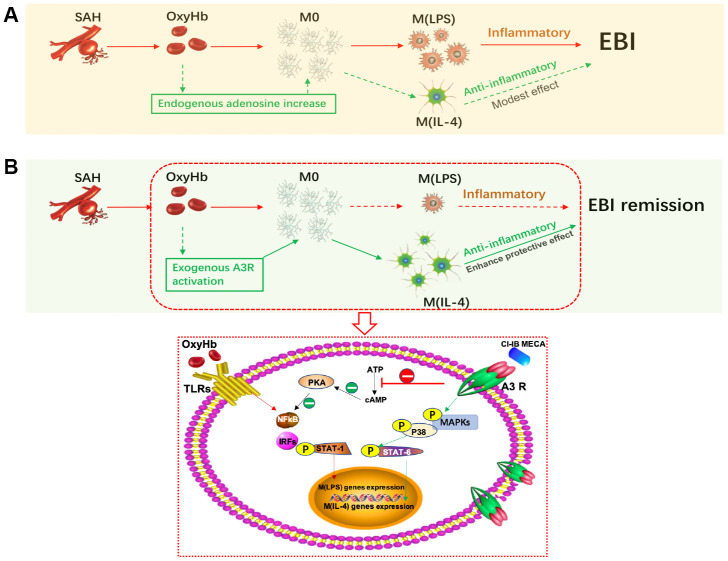
**Activation of adenosine A3 receptor (A3R) through the P38/STAT6 pathway modulates the phenotypic conversion of microglia and ameliorates early brain injury (EBI) after SAH.** (**A**) During the normal course of SAH, blood components promote the activation of resting microglia, mostly towards the M (LPS) phenotype, with a small proportion towards the M (IL-4) phenotype. The majority of M(LPS) microglia release excessive inflammatory cytokines when eliminating necrotic tissue debris, thus exacerbating neurological damage. Adenosine expression is upregulated in brain injury, which may promote the polarization of microglia towards the M(IL-4) phenotype to play a role in tissue repair. (**B**) Exogenous A3R agonists may contribute to microglial polarization towards the M(IL-4) phenotype, thereby providing neuroprotection and mitigating EBI. On the one hand, CI-IB-MECA can promote the transcription of M(IL-4) polarization-related genes by successively activating the MAPKs P38 and STAT6; on the other hand, it may also inhibit the PKA/ NF-κB pathway, which is associated with microglial M(LPS) polarization.

In this study, we found that the elderly rats showed obvious neurological impairment and microglial activation after SAH, accompanied by significant neuroinflammation (Figure 7). Age-related functional changes of microglia may partly explain the high mortality rate of SAH in the elderly and also suggest the importance of regulating microglia-mediated neuroinflammation. In this study, we showed that CI-IB-MECA suppressed the expression of IL-1β and TNF-α and elevated the expression of IL-4 and IL-10 both *in vivo* and *in vitro* by regulating microglial polarization towards the M(IL-4) phenotype. In contrast, either the A3R antagonist or siRNA eliminated the effect of CI-IB-MECA *in vivo* and *in vitro*. The results were consistent with our previous studies in adult models, which once again verified that CI-IB-MECA can reduce neuroinflammatory after SAH [24]. Long-term prevention of the M(LPS) phenotype and suppression of inflammation will not provide comprehensive beneficial effects, since the absence of M(LPS) microglia would hinder the clearance of cellular debris, thereby prolonging the inflammatory process [25]. However, inhibiting inflammation before it reaches its peak may prove to be an effective way to reduce brain damage. Indeed, in our study, we found that the neurobehavioral outcomes were improved 24 h after SAH when the aged rats were pretreated with CI-IB-MECA.

As G protein-coupled receptors, adenosine A3 receptors are widespread in the brains of a variety of species. Accordingly, accumulated research has focused on the neuroprotective function of A3R. Mice lacking A3R showed significant neurodegeneration induced by hypoxia or cerebral infarction [28, 45]. A3R is also essential for the chemotactic process extension and migration of microglia and play an anti-neuroinflammatory role [16, 46]. Since A3R is expressed both in the microglia and the neurons, previous studies showed that activation of A3R is involved in reducing neuronal injury [47, 48]. By reducing the death of neurons, microglia activation can also be alleviated. For the *in vivo* experiment, we found that the expression of A3R in the microglia rather than in the neurons increased after the administration of CI-IB-MECA. For the *in vitro* experiment, the death of neurons was directly induced by OxyHb, while more neurons died when co-cultured with activated microglia, indicating that microglia play a major role in the neuronal damage after SAH. Although CI-IB-MECA reduces the neuronal damage through both “direct” and “indirect” ways, it appears that inhibiting microglia activation can greatly reduce neuronal damage.

In the present study, we found that the expression of A3R was increased in the cerebral cortex in elderly rats after SAH or in microglia stimulated by OxyHb *in vitro*. This might be attributed to the increase in adenosine during conditions of increased metabolic demand or pathological states such as ischemia, pain and inflammation [49]. When necessary, ATP is increased and induces the A3R accumulation at the leading edge of immunocytes [50]. The increase in A3R and adenosine may be considered as an endogenous protective mechanism. In pathological or physiological conditions, the elevation of adenosine can activate A3R and also upregulate the receptor expression [47, 51–53]. But the underlying mechanism for A3R up-regulation can be complicated. Other studies demonstrated A3R recycling during which receptor experiences the internalization/ re-synthesis/re-expression when it binds to the ligand [54]. This specific metabolic pattern after activation of A3R may be an important reason for the up-regulation of ligand-induced receptor expression. Previous studies reported that CI-IB-MECA could induce desensitization and internalization of A3R, whereas receptor recycling was implicated in the resensitization of the receptor response [18, 55]. Therefore, it is highly possible that the relative decrease of A3R is caused by the binding of exogenous A3R ligands, while the increase of endogenous adenosine after SAH further induces the upregulation of A3R [53]. However, further verification is needed. As a highly selective A3 receptor agonist, CI-IB-MECA, reduced cerebral ischemic injury in rats and mediated neuroprotective effects via anti-inflammatory signaling [29, 56]. As suggested in this study, exogenous administration of A3 agonists can enhance the neuroprotective effects Figure 7. However, contrary to the above information, early studies also showed that IB-MECA, when acutely administered prior to ischemia, impaired post ischemic blood flow, increased infarct size and mortality, and exacerbated the loss of hippocampal neurons [22, 57]. It might be considered that this dual effect of activation of A3R depend on the cell type involved as well as on the duration of its activation [22]. It has also been reported that CI-IB-MECA can induce cell death and apoptosis, which may be related to drug concentration and cell type [58, 59]. It seems that CI-IB-MECA induced apoptosis may be related to a high concentration and may utilize different pathways by activating a specific extracellular receptor linked to some as yet unidentified signaling pathway, or, alternatively, it may directly enter cells to exert its toxicity [59]. It has been reported that the CI-IB-MECA at 10-100nM can reduce the death of the primary cultured neurons after ischemia-reperfusion injury, while the concentration above 10μM will result in dose-dependent oligodendrocyte death [28, 60]. Therefore, according to the literature, we used a relatively safe dose that can play a cellular protective role.

Although numerous signaling pathways are involved in immunocyte polarization, the foremost transcription factors include JAKs, STATs, and NF-κB. During immunocyte activation, phosphorylation of JAKs induces the dimerization and translocation of STAT1, which initiates M(LPS)-associated gene transcription and produces proinflammatory cytokines. Moreover, the activation of NF-κB p65 is characteristic of M(LPS) macrophage polarization [35]. On the other hand, STAT6 is essential for M(IL-4) polarization and induces expression of cell surface markers such as Arg-1, Fizz1, and CD206 and anti-inflammatory cytokines such as IL-10 [35]. Hence, we evaluated the activation of JAK1, STAT1, STAT6 and NF-κB p65 and found that the A3R agonist CI-IB-MECA suppressed NF-κB p65 and increased STAT6 activation (Figure 7). A3R is known to trigger a variety of intracellular signaling pathways regulating MAPK P38, ERK1/2 and PI3K/Akt [36]. Therefore, we hypothesized that there might exist crosstalks between P38 and STAT6 and indicated that CI-IB-MECA increased the expression of Arg-1 by sequentially activating P38 and STAT6. Other studies have also confirmed that STAT6, as a downstream signaling factor of P38, mediates IL-4-induced M(IL-4) phenotype polarization of macrophages [39]. Reportedly, STAT6 can also be expressed in neurons, and up-regulated SATA6 in neurons can enhance the release of inflammatory mediators that modulate the homeostatic microglial phenotype [37]. As the activators of STAT6, IL-13 and IL-4 can reduce microglia activation by directly inducing STAT6 phosphorylation in microglia and also by indirectly activating neuron STAT6 [37, 38]. Combining results *in vitro* and *in vivo*, we found that STAT6 activation caused by CI-IB-MECA may be mainly arisen in the microglia and that STAT6 phosphorylation was not induced by elevated IL-13 or IL-4.

In conclusion, the current work revealed that activation of A3R ameliorated neuronal apoptosis and improved neurobehavioral outcomes in aged rats by regulating the phenotypic conversion of microglia after SAH. The A3R agonist modulated microglial polarization and inflammatory cytokine release via the A3R/P38/STAT6 signaling pathway.

## MATERIALS AND METHODS

### Animals

Sprague–Dawley male rats (aged 20 months, 360-420 g) were obtained from the Animal Center of the Third Military Medical University, Chongqing, China. All experimental procedures were approved by the Animal Care and Use Committee of the Third Military Medical University and were in accordance with the guidelines of the National Institute of Health. All rats were housed in a light- and temperature-controlled environment with a 12-h/12-h light/dark cycle.

### Experimental SAH model

The experimental SAH model was implemented by endovascular puncture as previously described [61, 62]. The rats were anaesthetized by 1% sodium pentobarbital (40 mg/ kg, i.p). From the bifurcation of the common carotid artery, a sharpened 4-0 nylon suture was inserted into the internal carotid artery. After piercing the bifurcation of the middle cerebral artery and the anterior cerebral artery, the resistance decreased. Then, the nylon suture was quickly extracted to restore blood flow in the internal carotid artery to induce SAH. The sham animals had the same procedure without perforating their vessels. According to the blood clot in the subarachnoid space, the basilar cistern was partitioned into 6 segments with a score from 0 to 3 for each segment. When calculating the total scores, the scores of all areas were added (maximum SAH score =18). The SAH grade was evaluated by an investigator who was blinded to treatment groups and rats with a score of 8 or less were excluded.

### Cell culture and stimulation

Primary cortical neurons were prepared from embryos (E16) obtained from timed-pregnant Sprague-Dawley rats. Primary neurons were cultured as previously described [63]. BV-2 cells were resuspended in Dulbecco's modified Eagle's medium (DMEM) supplemented with 10% FBS and penicillin-streptomycin at 37° C in a humidified 5% CO_2_ atmosphere. To mimic SAH *in vitro*, cells were seeded in 24-well plates. Rats in the treatment groups were administered oxyhemoglobin (OxyHb, Sangon Biotech, China) at a concentration of 25 μM for 24 h.

### Experimental design and drug administration

In experiment 1, in order to study the changes in animal behavior and the phenotypes of microglia after SAH over time, the rats were divided into four groups at random (n=5 per group): the sham group, the SAH 24 h group, the SAH 3 d group, and the SAH 7 d group.

In experiment 2, the rats were randomly assigned to four groups (n=5 per group): the sham group, the SAH group, the SAH+A3R agonist (CI-IB-MECA) group, and the SAH+A3R antagonist (MRS1523) group. In this experiment, CI-IB-MECA (Abcam, USA) was dissolved in sterile saline and injected through the tail vein. To avoid inducing hypotension, CI-IB-MECA was administered twice (0.2 mg/kg per dose) at 165 min (first) and 15 min (second) before inducing SAH [64]. When needed, the A3R antagonist MRS1523 (1 mg/kg, i.p., Apexbio, USA) was administered to rats twice at 3 h and 1 h before inducing SAH [65]. Rats in the SAH groups were administered an equal volume of solvent. All rats were sacrificed 24 h after SAH.

In experiment 3, to further verify the role of the A3 agonist and antagonist in simulated SAH *in vitro* and to screen related pathways after A3R activation, BV-2 cells were divided into four groups: the sham group, the oxyhemoglobin (OxyHb) group, the OxyHb+CI-IB-MECA group, and the OxyHb+CI-IB-MECA+A3R siRNA group. CI-IB-MECA (50 nM) was administrated 1 h before OxyHb stimulation. In the siRNA groups, BV-2 cells were transfected with A3R siRNA or negative siRNA (Invitrogen, USA) at a final concentration of 60 nM for 48 h before treatment with OxyHb for 24 h.

In experiment 4, to further identify the role of P38 and STAT6 in A3R activation, BV-2 cells were assigned to five groups: the sham group, the CI-IB-MECA group, the CI-IB-MECA+A3R siRNA group, the CI-IB-MECA+P38 inhibitor (SB202190) group, and the CI-IB-MECA+STAT6 inhibitor (AS1517499) group. The cells were treated with CI-IB-MECA (50 nM) for 1 h. In the siRNA groups, A3R siRNA was administered 48 h before initiating the treatment with CI-IB-MECA. In the other groups, the cells were treated with the P38 inhibitor SB202190 (20 μM, Abcam, USA) or the STAT6 inhibitor AS1517499 (1 μM, AXON Medchem, Netherlands) for 1 h before CI-IB-MECA administration.

In experiment 5, to determine the effect of blocking the A3R/ P38/STAT6 pathway on the release of cytokines from microglia, based on the findings of experiment 3, the P38 inhibitor SB202190 or STAT6 inhibitor AS1517499 were also used to pretreat cells before CI-IB-MECA administration. BV-2 cells were assigned to one of six groups: the sham group, the oxyhemoglobin (OxyHb) group, the OxyHb+CI-IB-MECA group, the OxyHb+CI-IB-MECA+A3R siRNA group, the OxyHb+CI-IB-MECA+SB202190 group, and the OxyHb+CI-IB-MECA+AS1517499 group. The dose and time were compared with those of the above experiments.

### Neurological score

The modified Garcia scoring system was used to evaluate neurological scores at 24 h, 3 d and 7 d after SAH [66]. Briefly, six tests on a scale of 0 to 3 or 1 to 3 were included in the assessment: spontaneous movement, symmetry of limb movements, forelimb extension, climbing ability, proprioception of the body and response to vibration stimuli. The scores were assessed by an observer who was blind to treatment conditions.

### Microglia and neuron co-culture

In the co-culture experiment shown in Figure 4A, neurons and microglia were co-cultured in a dish with transwell (Corning, USA). To examine the “direct” effects of CI-IB-MECA, primary neurons were incubated alone with OxyHb in the presence or absence of CI-IB-MECA and/or siRNA for A3R for 48h. To examine the “indirect” effects of CI-IB-MECA, primary neurons were cultured in the lower compartment and BV2 microglia were cultured on transwell inserts. Before the co-culture, microglia and neurons were separately incubated with OxyHb for 24 h. Finally, the preprocessed microglia and neureons were co-cultured for another 24 h. CI-IB-MECA, siRNA, P38 and STAT6 inhibitor were preincubated microglia in each group at the dose described above.

### Western blot analysis

The cerebral cortex tissues with blood clots or cultured cells were collected and lysed in 20 mM Tris (Beyotime, China). Western blotting was conducted following the standard protocol. The following primary antibodies were used: anti-induced nitric-oxide synthase (iNOS) antibody, anti-adenosine A3 receptor antibody, anti-liver arginase antibody (Arg-1), anti-cleaved caspase 3 antibody, anti-IL-4 antibody, anti-IL-13 antibody, anti-p-P38 antibody (Thr180/Tyr182), anti-p-STAT6 antibody (Tyr641), anti-p-JAK1 antibody (Tyr1022/Tyr1023), anti-p-STAT1 antibody (Tyr701), and anti-p-NF-κB p65 antibody (Ser276) (Abcam, USA). Anti-tubulin rabbit antibody and goat anti-rabbit and rabbit anti-goat secondary antibodies were used (Beyotime, China). The blotted protein bands were quantified by Image J software (National Institutes of Health, USA). The density values are shown as the target protein OD/β-tubulin OD ratio.

### Immunofluorescence staining

Immunofluorescence staining was performed according our previous study [67]. Brain sections from cerebral cortex and cultured cells on coverslips were fixed in 4% paraformaldehyde. Samples were blocked with 5% BSA prior to incubation with primary antibody overnight with the following antibodies: the M (LPS) marker iNOS or the M (IL-4) marker Arg-1, the microglial marker F4/80 and the neuron marker NeuN (Abcam, USA). After washing with PBS, sections or slides of cells were then incubated with the appropriate secondary antibodies, Alexa Fluor 488 goat anti-rabbit IgG and Alexa Fluor 594 goat anti-Rat IgG (Abcam, USA), for 1 h at room temperature. After washing, the samples were restained with 4',6-diamidino-2-phenylindole (DAPI) for 12 min before mounting. Five random fields of each sample were observed and photographed with a fluorescence microscope (Olympus BX51TRF, Olympus Co., Tokyo, Japan).

### TUNEL staining

The cerebral cortex tissues with blood clots were collected. TUNEL staining was performed according to the manufacturer’s instructions (One Step TUNEL Apoptosis Assay Kit, Beyotime, China). Then, the samples were counterstained with NeuN (Abcam, USA). The apoptotic cells were identified, counted, and analyzed by a researcher who was blind to the groupings.

### 3-(4,5-Dimethylthiazol-2-yl)-2,5-diphenyltetrazolium bromide (MTT) assay

MTT Assay Kit (Abcam, USA) were used to evaluate neuronal viability. Cells were digested and inoculated on culture plate. After incubating in 5% CO_2_ at 37° C, MTT was added into cells and continued to culture. Adding DMSO, the absorbance (OD value) was measured at 490 nm by an enzyme-linked immunosorbent assay.

### Real-time PCR

RNA extracted by an RNA extraction kit (Fastagen, China) was reverse transcribed into cDNA with PrimeScriptTM RT master mix (TaKaRa, China). Quantitative real-time PCR analysis was performed on an iCycler iQ Real-Time PCR Detection System using a SYBR Premix ExTaq kit (TaKaRa, China). GAPDH was used for normalization. The amplification conditions were 95° C for 30 s, 35 cycles of denaturation at 95° C for 5 s, and an annealing step at 60° C for 20 s. The sequences of the primer pairs were as follows: IL-1β: GCAACTGTTCCTGAACTCAACT (forward), GGATTTTGTCGTTGCTTGTCTC(reverse); TNF-α: CTGTAGCCCACGTCGTAGC (forward), AGACATCTTCAGCAGCCTTGTGAG (reverse); IL-4: ACTTGAGAGAGATCATCGGCA (forward), TTGCGAAGCACCCTGGAAG (reverse); and IL-10: CCAAGCCTTATCGGAAATGA (forward), CAAGGCTTGGCAACCCAAGTA (reverse).

### Enzyme-linked immunosorbent assay

Quantification of the protein levels of IL-1β, TNF-α, IL-4 and IL-10 was performed by the enzyme-linked immunosorbent assay (ELISA). According to the manufacturer’s instructions, homogenates of the cortex or cultured medium of cells were prepared for detection (Boster, China). Values were expressed as pg/mg or pg/ml. The results were normalized to protein levels.

### Statistics

All statistical analyses were performed using SPSS software. All data are presented as the mean ± SD. The results were analyzed with one-way ANOVA, followed by Tukey’s post hoc test to assess multiple comparisons. *P* < 0.05 was considered statistically significant.
